# Knowledge, attitude, and practice toward ultrasound screening for breast cancer among women

**DOI:** 10.3389/fpubh.2024.1309797

**Published:** 2024-05-24

**Authors:** Shaozhong Liu, Shukai Zheng, Mengzhen Qin, Yifeng Xie, Kun Yang, Xiaozhen Liu

**Affiliations:** ^1^Department of Ultrasound Imaging, Zhongshan City People’s Hospital, Zhongshan, China; ^2^Department of Breast Surgery, Zhongshan City People’s Hospital, Zhongshan, China

**Keywords:** knowledge, attitude, practice, breast cancer, screening, ultrasound, cross-sectional study

## Abstract

**Background:**

Several obstacles can hinder breast cancer screening. This study aimed to investigate the knowledge, attitude, and practice (KAP) toward ultrasound screening for breast cancer in women.

**Methods:**

This cross-sectional study recruited women who visited the breast specialist clinic of Zhongshan City People’s Hospital (a tertiary hospital) between August 2022 and April 2023 through convenience sampling. KAP scores ≥70% were considered adequate.

**Results:**

This study enrolled 501 participants. The mean knowledge, attitude, and practice levels were 8.56 ± 1.81/12 (possible range 0–12, 71.33%), 29.80 ± 2.71 (possible range 8–40, 74.50%), and 32.04 ± 3.09 (possible range 8–40, 80.10%). Senior high school education (vs. junior high school and below, coefficient = 1.531, 95%CI: 1.013–2.312, *p* = 0.044), bachelor’s education and above (vs. junior high school and below, coefficient = 5.315, 95%CI: 3.546–7.966, *p* < 0.001), housewife or unemployed (vs. employed, coefficient = 0.671, 95%CI: 0.466–0.966, *p* = 0.032), and a history of breast ultrasound (vs. no, coefficient = 1.466, 95%CI: 1.121–1.917, *p* = 0.005) were independently and positively associated with knowledge. Knowledge (coefficient = 1.303, 95%CI: 1.100–1.544, *p* = 0.002) and monthly income >10,000 (vs. <5,000, coefficient = 4.364, 95%CI: 1.738–10.956, *p* = 0.002) were independently and positively associated with attitude. Only attitude (coefficient = 1.212, 95%CI: 1.096–1.340, *p* < 0.001) was independently and positively associated with the practice. A structural equation modeling (SEM) analysis was used to estimate causality among KAP dimensions, showing that knowledge directly influenced attitude (*β* = −1.090, *p* = 0.015), knowledge did not directly influence practice (*β* = −0.117, *p* = 0.681) but had an indirect influence (*β* = 0.826, *p* = 0.028), and attitude directly influenced practice (*β* = −0.757, *p* = 0.016).

**Conclusion:**

Women in Zhongshan City had good knowledge, favorable attitudes, and active practice toward breast ultrasound screening for breast cancer. Women’s characteristics associated with a poorer KAP were identified, allowing for more targeted interventions.

## 1 Introduction

Breast cancer is the most common cancer diagnosed in women worldwide (1, 2), with 2,261,419 new cases in 2020 (estimated) and 684,996 deaths (3). The risk factors include gene mutations and polymorphisms, older age, no pregnancies, hormone exposure, lifestyle, and radiation exposure (4, 5). Despite the high incidence and mortality, breast cancer survivors in the United States of America display a 5-year survival of 99% for women with localized disease, 85% for regional disease, and 27% for women with distant metastases (6).

Many patients are asymptomatic until reaching the advanced or metastatic stage. Hence, breast cancer is mainly detected through screening for abnormalities, including palpable breast mass, palpable axillary mass, nipple discharge, skin changes, asymmetric changes, or pain (5). Screening for breast cancer refers to testing performed in asymptomatic women to detect the disease early to decrease morbidity and/or mortality (5). The 15-year absolute reduction in BC-related mortality with mammography is 40.6 deaths per 100,000 women aged 40–49, 61.7 deaths per 100,000 women ≥50 years of age, and 211.8 deaths per 100,000 women aged 60–69 (7, 8). Still, the impact on overall survival remains controversial (8, 9).

Screening modalities include breast self-examination, physical examination, and imaging, with mammography being the screening modality of choice for the early detection of breast cancer (5). Ultrasound is also a modality of choice for examining breast symptoms like a mass or nipple discharge (5). Ultrasound can be used as an adjunct to mammography for detecting breast cancer, especially in women with dense breasts (10–14). In women with dense breasts, the sensitivity of mammography is 50%, while the sensitivity of mammography plus ultrasound is 77.5% (10, 15). Still, breast ultrasound screening can be performed stand-alone and is popular in some countries, with many women living in remote areas (16). Indeed, suitcase-sized portable ultrasound systems are available and have no special power or occupational exposure requirements, while portable mammography systems are not available. In addition, mammography requires radiation safety procedures.

Because Chinese women have denser breasts than Westerners (17) and because breast ultrasound is non-invasive and inexpensive, it is a popular BC screening modality in China (18). A central feature of breast cancer screening programs is their voluntary aspect (16, 19). The women’s health literacy and beliefs will influence how they participate in breast cancer screening. Hence, Chinese women from the general population may lack the proper knowledge and awareness of ultrasound screening for breast cancer. In addition, several programs offer different screening modalities, and the women’s choices can influence the screening outcomes. The knowledge, attitude, and practice (KAP) methodology provides qualitative and quantitative data about the gaps, misunderstandings, misconceptions, and barriers toward the optimal implementation of a given healthcare concept in a given population (20, 21).

No data on the KAP toward breast cancer ultrasound screening in continental China are available. Still, previous studies reported highly variable KAP toward breast cancer screening in different countries (22–25). Breast screening services and programs vary widely among countries with different characteristics, socioeconomic characteristics, customs, patient education, and healthcare systems, and the results of KAP studies cannot be extrapolated to other countries or sometimes regions. Therefore, it is crucial to identify the obstacles hindering breast cancer screening in China to adopt more effective methods to promote screening. Therefore, this study aimed to assess the KAP of women toward ultrasound screening for breast cancer. The results could be used to design and implement educational and motivational interventions to improve the KAP toward breast cancer screening in Chinese women.

## 2 Methods

### 2.1 Study design and participants

This cross-sectional study was conducted at Zhongshan City People’s Hospital between August 2022 and April 2023. The participants were women recruited by convenience sampling. The study was approved by the Medical Ethics Committee of Zhongshan People’s Hospital. All participants signed the informed consent form.

The inclusion criteria were (1) Han nationality and (2) ≥18 years of age. The exclusion criteria were (1) cognitive impairment, (2) communication disorder, or (3) not completing the questionnaire.

### 2.2 Questionnaire

The questionnaire was designed according to previous studies (26, 27). The questionnaire was reviewed by two senior experts. The questionnaire was pre-tested by 53 women, and Cronbach’s α coefficient value was 0.782 (i.e., acceptable internal consistency).

The final questionnaire included four dimensions: demographic characteristics, knowledge dimension (including knowledge of breast cancer and breast ultrasound), attitude dimension, and practice dimension (Supplementary material). The basic characteristics were covered by nine items. The knowledge part included 12 items; correct answers were scored 1 point, and wrong/unclear answers were scored 0 points, with a theoretical score range of 0–12 points. The attitude part consisted of eight items, using a 5-point Likert scale, from very positive (5 points) to very negative (1 point). The total score ranged from 8 to 40 points. The practice part included eight items, using a 5-point Likert scale, from very positive (5 points) to very negative (1 point). The total score ranged from 8 to 40 points.

The questionnaires were distributed to the participants in breast specialist clinics. Five doctors and nurses were responsible for promoting and distributing the questionnaires were trained for this study and acted as research assistants.

### 2.3 Sample size

The formula


$$n={\left(\frac{Z_{1-\alpha /2}}{\delta }\right)}^{2}\times p\times \left(1-p\right)$$

can be used to calculate the sample size of cross-sectional surveys. In the formula, “*n*” represents the sample size for each group, “*α*” represents the type I error, which is typically set at 0.05, *Z*_1-α/2_ = 1.96, δ represents the allowable error, typically set at 0.05, and “*p*” is set at 0.5 (as setting it at 0.5 maximizes the value and ensures a sufficiently large sample size). Hence, the calculated sample size was 384. Considering an estimated questionnaire response rate of 80%, 480 valid questionnaires were needed.

### 2.4 Statistical analysis

SPSS 26.0 (IBM Corporation, Armonk, NY, United States) was used for analysis. The continuous variables were presented as mean ± standard deviation (SD) and analyzed using Student’s *t*-test (two groups) or one-way ANOVA (more than two groups). Categorical variables were expressed as *n* (%). The Spearman analysis was used to analyze the correlation of knowledge, attitude, and practice scores. The variables with *p* < 0.10 in the univariable analyses were entered in the multivariable linear regression analyses to determine the factors independently associated with KAP. A structural equation modeling (SEM) is a statistical statement of the relationship between variables, sometimes called a path diagram, and is a specific representation of the model in a graphical manner. The relationship between each latent variable should be supported theoretically or proved in practice, and finally, a research framework diagram. SEM was employed to test the following hypotheses: (1) knowledge had impacts on attitude; (2) knowledge had impacts on practice; (3) attitude had impacts on practice. Confirmatory factor analysis and model fitting were evaluated using the following indices: CFI (comparative fit index), IFI (incremental fit index), TLI (Tucker–Lewis index), RMSEA (root mean square error of approximation), and CMIN/DF (chi-square value/degrees of freedom). Two-sided *p*-values <0.05 were regarded as statistically significant.

## 3 Results

### 3.1 Characteristics of the study population

The study enrolled 514 participants, with two persons under 18 years of age and 11 persons belonging to ethnic minorities. Finally, 501 valid questionnaires were valid. Most participants were 30–39 (42.32%), living in urban areas (52.3%), had a bachelor’s degree or above (62.87%), with children (54.09%), employed (86.03%), with monthly income <5,000 (53.49%), without a family history of breast cancer (96.41%), and underwent a breast ultrasound (72.06%) (Table 1).

**Table 1:** Characteristics of the participants.

Variables	*n* (%)	Knowledge	*P*	Attitude	*P*	Practice	*P*
Age, years		8.56 ± 1.81	0.767	29.8 ± 2.71	0.212	32.04 ± 3.09	0.974
<30	170 (33.93)	8.62 ± 1.67		30.01 ± 2.60		31.99 ± 3.27	
30–39	212 (42.32)	8.56 ± 1.99		29.82 ± 2.88		32.08 ± 3.18	
≥40	119 (23.75)	8.49 ± 1.70		29.48 ± 2.52		32.03 ± 2.64	
Residence			<0.001		0.009		0.659
Urban	262 (52.3)	9.00 ± 1.58		30.1 ± 2.84		32.13 ± 3.15	
Non-urban	239 (47.7)	8.08 ± 1.93		29.48 ± 2.52		31.94 ± 3.02	
Marital status			0.028		0.054		0.662
Unmarried	113 (22.55)	8.94 ± 1.68		30.28 ± 2.9		32.19 ± 3.31	
Married	345 (68.86)	8.45 ± 1.89		29.62 ± 2.64		32.03 ± 3.08	
Divorced or widowed	43 (8.58)	8.49 ± 1.35		30.00 ± 2.63		31.72 ± 2.54	
Education			<0.001		0.003		0.253
Junior high school and below	93 (18.56)	6.76 ± 1.92		29.04 ± 2.61		31.56 ± 3.07	
Senior high school	93 (18.56)	8.05 ± 1.65		29.74 ± 2.40		31.94 ± 3.01	
Bachelor and above	315 (62.87)	9.25 ± 1.36		30.04 ± 2.78		32.21 ± 3.10	
Fertility status			<0.001		0.023		0.433
Childbearing	271 (54.09)	8.23 ± 1.99		29.53 ± 2.61		31.96 ± 3.04	
No pregnancy	192 (38.32)	9.02 ± 1.54		30.23 ± 2.74		32.00 ± 3.20	
Pregnancy but not given birth	38 (7.58)	8.71 ± 1.25		29.61 ± 2.99		32.76 ± 2.79	
Working status			<0.001		0.201		0.307
Employed	431 (86.03)	8.77 ± 1.67		29.85 ± 2.73		32.1 ± 3.06	
Housewife or unemployed	70 (13.97)	7.3 ± 2.16		29.5 ± 2.56		31.66 ± 3.26	
Monthly income, CNY			<0.001		0.012		0.302
<5,000	268 (53.49)	8.26 ± 1.91		29.57 ± 2.6		31.91 ± 2.89	
5,000–10,000	195 (38.92)	8.89 ± 1.57		29.84 ± 2.62		32.11 ± 3.03	
>10,000	38 (7.58)	9.03 ± 1.95		31.26 ± 3.41		32.55 ± 4.43	
Family history of breast cancer			0.473		0.915		0.899
Yes	18 (3.59)	8.39 ± 1.75		29.89 ± 2.59		32 ± 2.74	
No	483 (96.41)	8.57 ± 1.82		29.8 ± 2.71		32.04 ± 3.1	
Breast ultrasound			0.005		0.031		0.220
Yes	361 (72.06)	8.72 ± 1.69		29.96 ± 2.65		32.17 ± 3.08	
No	140 (27.94)	8.16 ± 2.05		29.39 ± 2.82		31.7 ± 3.07	

The confirmatory factor analysis showed CFI = 0.816 (>0.8 is good), IFI = 0.826 (>0.8 is good), RMSEA = 0.025 (<0.08 is good), and CMIN/DF = 1.306.

### 3.2 Knowledge

The knowledge score was 8.56 ± 1.81 (0–12, 71.33%). Better knowledge was observed in urban residents (*p* < 0.001), unmarried women (*p* = 0.028), with higher education (*p* < 0.001), women without children (*p* < 0.001), employed (*p* < 0.001), with higher income (*p* < 0.001), and already underwent breast ultrasound (*p* = 0.005) (Table 1). The items with scores <70% were K2 (39.52%; “The age at which women are susceptible to breast cancer”), K7 (51.50%; “Breast ultrasound has radiation like an X-ray”), K3 (53.69%; “The optimal interval for regular breast cancer screening in healthy women”), K10 (54.69%; “Breast ultrasound is only important for women with a family history of breast cancer”), and K4 (57.88%; “The pre-symptoms of breast cancer”) (Table 2).

**Table 2:** Knowledge dimension.

Knowledge	*N* (%)	
	Wrong	True
1. Breast cancer is the most common malignant tumor that seriously threatens women’s health.	62 (12.38)	439 (87.62)
2. The age at which women are susceptible to breast cancer.	303 (60.48)	198 (39.52)
3. The optimal interval for regular breast cancer screening in healthy women.	232 (46.31)	269 (53.69)
4. The pre-symptoms of breast cancer.	211 (42.12)	290 (57.88)
5. Breast cancer can be detected early through breast screening.	56 (11.18)	445 (88.82)
6. You have heard of a test called breast ultrasound	62 (12.38)	439 (87.62)
7. Breast ultrasound has radiation like an X-ray.	243 (48.5)	258 (51.5)
8. Breast ultrasound can screen for breast cancer.	139 (27.74)	362 (72.26)
9. Breast ultrasound is only important for women over 50.	121 (24.15)	380 (75.85)
10. Breast ultrasound is only important for women with a family history of breast cancer.	227 (45.31)	274 (54.69)
11. Besides the doctor or their palpation examination, women should also have a breast ultrasound.	45 (8.98)	456 (91.02)
12. Women aged 41–70 should have a breast ultrasound at least once a year.	20 (3.99)	481 (96.01)

### 3.3 Attitude

The attitude score was 29.80 ± 2.71 (5–40, 74.50%). A better attitude was observed in urban residents (*p* = 0.009), higher education (*p* = 0.003), without children (*p* = 0.023), higher income (*p* = 0.012), and already underwent breast ultrasound (*p* = 0.031) (Table 1). Table 3 shows the distribution of the responses to each attitude item.

**Table 3:** Attitude dimension.

	Strongly agree	Agree	Neutrality	Disagree	Strongly disagree
1. I was very afraid that I would get breast cancer.	247 (49.3)	142 (28.34)	107 (21.36)	3 (0.6)	2 (0.4)
2. I think early detection, early diagnosis, and early treatment are very important for breast cancer prevention.	427 (85.23)	73 (14.57)	1 (0.20)	0	0
3. I do a breast ultrasound only because my doctor has already booked an appointment.	98 (19.56)	56 (11.18)	106 (21.16)	190 (37.92)	51 (10.18)
4. If the doctor did not recommend it, I would not do a breast ultrasound.	38 (7.58)	40 (7.98)	70 (13.97)	243 (48.5)	110 (21.96)
5. A breast ultrasound changes my chances of finding a lump before I can feel it.	245 (48.9)	136 (27.15)	50 (9.98)	68 (13.57)	2 (0.4)
6. Having a breast ultrasound once a year will make me feel very at ease.	293 (58.48)	174 (34.73)	34 (6.79)	0	0
7. I think breast ultrasound can detect breast cancer that cannot be detected by mammograms alone.	94 (18.76)	99 (19.76)	215 (42.91)	90 (17.96)	3 (0.6)
8. I want to know more about breast cancer and breast screening.	262 (52.3)	195 (38.92)	0	43 (8.58)	1 (0.2)

### 3.4 Practice

The practice score was 32.04 ± 3.09 (5–40, 80.10%) (Table 1). Table 4 shows the distribution of the responses to the practice items.

**Table 4:** Practice dimension.

	Strongly agree	Agree	Neutrality	Disagree	Strongly disagree
1. If there is a seminar related to breast cancer and screening, I would like to attend.	164 (32.73)	236 (47.11)	97 (19.36)	4 (0.80)	0
2. I want to know if I’m at high risk for breast cancer.	202 (40.32)	249 (49.70)	44 (8.78)	5 (1.00)	1 (0.2)
3. I plan to have a breast ultrasound at least once a year.	250 (49.90)	202 (40.32)	48 (9.58)	1 (0.20)	0
4. If a friend/relative recommends a breast ultrasound to me, I will do it	254 (50.70)	199 (39.72)	46 (9.18)	2 (0.40)	0
5. If I know someone has been diagnosed with breast cancer, I will make an appointment for a breast ultrasound as soon as possible.	139 (27.74)	196 (39.12)	112 (22.36)	54 (10.78)	0
6. If I am not unwell, I will not go for a breast ultrasound.	39 (7.78)	102 (20.36)	110 (21.96)	196 (39.12)	54 (10.78)
7. If the last breast ultrasound test is negative, I will relax my vigilance for breast diseases.	40 (7.98)	95 (18.96)	101 (20.16)	221 (44.11)	44 (8.78)
Between the two screenings, I will always pay attention to the breast glands and seek medical attention in time if abnormalities are found.	271 (54.09)	196 (39.12)	33 (6.59)	1 (0.20)	0

### 3.5 Correlations

The knowledge scores were correlated to the attitude (*r* = 0.231, *p* < 0.001) and practice (*r* = 0.121, *p* < 0.001) scores. The attitude scores were correlated to the practice scores (*r* = 0.166, *p* < 0.001) (Table 5).

**Table 5:** Correlation analysis.

	Knowledge	Attitude	Practice
Knowledge	1		
Attitude	0.231 (*P* < 0.001)	1	
Practice	0.121 (*P* < 0.001)	0.166 (*P* < 0.001)	1

### 3.6 Multivariable analyses

Multivariate linear regression analysis showed senior high school education (vs. junior high school and below, coefficient = 1.531, 95%CI: 1.013–2.312, *p* = 0.044), bachelor’s education and above (vs. junior high school and below, coefficient = 5.315, 95%CI: 3.546–7.966, *p* < 0.001), housewife or unemployed (vs. employed, coefficient = 0.671, 95%CI: 0.466–0.966, *p* = 0.032), and a history of breast ultrasound (vs. no, coefficient = 1.466, 95%CI: 1.121–1.917, *p* = 0.005) were independently and positively associated with knowledge (Table 6). Knowledge (coefficient = 1.303, 95%CI: 1.100–1.544, *p* = 0.002) and monthly income >10,000 (vs. <5,000, coefficient = 4.364, 95%CI: 1.738–10.956, *p* = 0.002) were independently and positively associated with attitude (Table 7). Only attitude (coefficient = 1.212, 95%CI: 1.096–1.340, *p* < 0.001) was independently and positively associated with practice (Table 8). Hence, those factors are independently associated with a better KAP toward breast ultrasound screening. Still, multivariable analyses of cross-sectional data cannot provide causality.

**Table 6:** Univariable and multivariable logistic regression analysis for knowledge score.

Knowledge	Univariable analysis		Multivariable analysis	
	Coefficient (95%CI)	*P*	Coefficient (95%CI)	*P*
Age				
<30	REF			
30–39	0.829 (0.603,1.141)	0.249		
≥40	1.031 (0.712,1.493)	0.873		
Residence				
Urban	REF		REF	
Non-urban	0.453 (0.346,0.592)	<0.001	0.958 (0.724,1.267)	0.763
Marital status				
Unmarried	REF		REF	
Married	0.611 (0.438,0.853)	0.004	0.897 (0.658,1.224)	0.494
Divorced or widowed	0.761 (0.438,1.321)	0.330	1.181 (0.724,1.926)	0.505
Education				
Junior high school and below	REF		REF	
Senior high school	1.676 (1.127,2.492)	0.011	1.531 (1.013,2.312)	0.044
Bachelor and above	6.203 (4.507,8.537)	<0.001	5.315 (3.546,7.966)	<0.001
Fertility status				
Childbearing	REF		REF	
No pregnancy	2.023 (1.519,2.694)	<0.001	0.887 (0.657,1.196)	0.431
Pregnancy but not given birth	1.272 (0.752,2.151)	0.370	0.748 (0.463,1.210)	0.238
Working status				
Employed	REF		REF	
Housewife or unemployed	0.376 (0.255,0.556)	<0.001	0.671 (0.466,0.966)	0.032
Monthly income, CNY				
<5,000	REF		REF	
5,000–10,000	1.902 (1.428,2.533)	<0.001	1.278 (0.983,1.663)	0.068
>10,000	1.608 (0.949,2.726)	0.078	0.978 (0.606,1.579)	0.929
Family history of breast cancer				
Yes	0.820 (0.389,1.727)	0.601		
No	REF			
Underwent a breast ultrasound				
Yes	1.588 (1.169,2.157)	0.003	1.466 (1.121,1.917)	0.005
No	REF		REF	

**Table 7:** Univariable and multivariable logistic regression analysis for attitude score.

Attitude	Univariable analysis		Multivariable analysis	
	Coefficient (95%CI)	*P*	Coefficient (95%CI)	*P*
Knowledge score	1.382 (1.192,1.602)	0.002	1.303 (1.100,1.544)	0.002
Age				
<30	REF			
30–39	0.831 (0.481,1.436)	0.506		
≥40	0.590 (0.313,1.114)	0.104		
Residence				
Urban	REF			
Non-urban	0.541 (0.337,0.868)	0.011	0.778 (0.454,1.332)	0.360
Marital status				
Unmarried	REF			
Married	0.515 (0.290,0.915)	0.024	0.650 (0.358,1.181)	0.158
Divorced or widowed	0.753 (0.292,1.946)	0.558	1.085 (0.423,2.780)	0.866
Education				
Junior high school and below	REF		REF	
Senior high school	2.012 (0.928,4.359)	0.076	1.661 (0.760,3.633)	0.204
Bachelor and above	2.722 (1.461,5.072)	0.002	0.971 (0.433,2.179)	0.943
Fertility status				
Childbearing	REF			
No pregnancy	2.017 (1.225,3.320)	0.006	1.583 (0.889,2.817)	0.119
Pregnancy but not given birth	1.081 (0.433,2.699)	0.868	0.837 (0.332,2.110)	0.706
Working status				
Employed	REF			
Housewife or unemployed	0.704 (0.355,1.396)	0.314		
Monthly income, CNY				
<5,000	REF		REF	
5,000–10,000	1.315 (0.802,2.157)	0.277	0.990 (0.596,1.644)	0.970
>10,000	5.452 (2.192,13.558)	<0.001	4.364 (1.738,10.956)	0.002
Family history of breast cancer				
Yes	1.094 (0.305,3.923)	0.890		
No	REF			
Underwent a breast ultrasound				
Yes	1.765 (1.042,2.991)	0.035	1.580 (0.939,2.657)	0.085
No	REF		REF	

**Table 8:** Univariable and multivariable logistic regression analysis for practice score.

Practice	Univariable analysis		Multivariable analysis	
	Coefficient (95%CI)	*P*	Coefficient (95%CI)	*P*
Knowledge score	1.223 (1.031,1.450)	0.021	1.118 (0.919,1.359)	0.265
Attitude score	1.233 (1.118,1.361)	<0.001	1.212 (1.096,1.340)	<0.001
Age				
<30	REF			
30–39	1.096 (0.587,2.049)	0.773		
≥40	1.046 (0.506,2.162)	0.902		
Residence				
Urban	REF			
Non-urban	0.832 (0.483,1.430)	0.504		
Marital status				
Unmarried	REF			
Married	0.855 (0.443,1.650)	0.640		
Divorced or widowed	0.628 (0.212,1.864)	0.401		
Education				
Junior high school and below	REF			
Senior high school	1.457 (0.600,3.540)	0.405	1.203 (0.499,2.900)	0.681
Bachelor and above	1.916 (0.938,3.915)	0.074	1.290 (0.587,2.836)	0.526
Fertility status				
Childbearing	REF			
No pregnancy	1.038 (0.586,1.838)	0.899		
Pregnancy but not given birth	2.226 (0.779,6.358)	0.135		
Working status				
Employed	REF			
Housewife or unemployed	0.642 (0.294,1.403)	0.266		
Monthly income, CNY				
<5,000	REF			
5,000–10,000	1.224 (0.692,2.166)	0.486		
>10,000	1.901 (0.664,5.437)	0.231		
Family history of breast cancer				
Yes	1.598 (0.876,2.916)	0.127		
No	REF			
Underwent a breast ultrasound				
Yes	0.961 (0.225,4.111)	0.958		
No	REF			

### 3.7 Structural equation modeling

**Figure 1:**
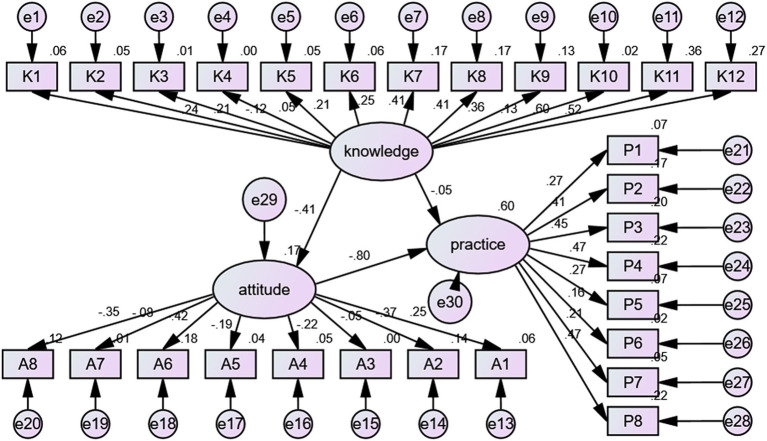
Schematic representation of the structural equation modeling analysis.

The SEM analyses are surrogates for causality, providing an estimation of causality based on prespecified hypotheses and a graphical model of the possible relationships among variables (28–30). Table 9 shows that the goodness-of-fit of the SEM analysis was good/excellent. SEM analysis showed knowledge directly influenced attitude (*β* = −1.090, *p* = 0.015). Knowledge did not directly influence practice (*β* = −0.117, *p* = 0.681) but had an indirect influence (*β* = 0.826, *p* = 0.028). Attitude directly influenced practice (*β* = −0.757, *p* = 0.016) (Table 10 and Figure 1).

**Table 9:** Goodness of fit indices of the SEM analysis.

Index	Reference standards	Values
CMIN/DF	1–3 Excellent, 3–5 Good	1.430
RMSEA	<0.08 Good	0.029
IFI	>0.8 Good	0.794
TLI	>0.8 Good	0.766
CFI	>0.8 Good	0.785

**Table 10:** The direct and indirect estimates of SEM.

Model paths	Direct effect		Indirect effect	
	β (95% CI)	*P*	β (95% CI)	*P*
Attitude ← knowledge	−1.090 (−3.428--0.379)	0.015		
Practice ← knowledge	−0.117 (−1.268–0.556)	0.681	0.826 (0.131–2.646)	0.028
Practice ← attitude	−0.757 (−1.672--0.291)	0.016		

## 4 Discussion

The results suggest that women in Zhongshan had adequate knowledge, favorable attitudes, and active practice toward breast ultrasound, but gaps were still observed, and they should be the focus of future educational and motivational interventions. This study also identified broad categories of women in Zhongshan who might benefit more from such interventions. The SEM analysis showed knowledge influenced attitude but not practice, while attitude influenced practice. This study may provide a basis for whether to carry out educational intervention and improve women’s KAP for ultrasound breast cancer screening in Zhongshan. Of course, additional studies are necessary to determine whether the results are similar in other areas in China.

Breast cancer screening is voluntary and requires the willingness and participation of the women (5). Therefore, basic knowledge and good attitudes toward breast cancer and breast cancer screening are essential for participation in screening. Several countries have screening programs for breast cancer, with letters sent to women reaching a certain age inviting them to participate, followed by letters at regular intervals to remind them to participate (16, 19). The KAP toward breast cancer screening varies widely among countries with different characteristics, customs, and healthcare systems (22–25). A study in Saudi Arabia showed that most women had poor knowledge of breast cancer screening and screening methods, and social media was their main source of information (22). Saudi Arabia offers a free breast cancer screening program to all women above age 40 (31), and access to breast cancer screening should not be an impediment to knowledge acquisition. In Jordan, most women had adequate knowledge of breast cancer screening, but the participation rates in screening mammography were low (23). Jordan also has a breast cancer screening program. Heena et al. (24) showed that female healthcare professionals (including physicians, nurses, and other healthcare professionals) in Saudi Arabia, Pakistan, and the United Arab Emirates had lower-than-expected knowledge about breast cancer screening, considering their educational background and medical training. Qatari women also have poor knowledge and participation in breast cancer screening (25), despite the fact that Qatar has a program inviting women ages 45–69 to undergo breast cancer screening every 3 years (32). Healthcare professionals are first-line sources of reliable health-related knowledge for many individuals (33, 34), highlighting the need for proper training and knowledge.

The Chinese economy is developing at a rapid pace, and society is evolving at a similarly fast pace. Hence, improving breast cancer prevention and control is a crucial public health issue. The breasts of Chinese women are generally smaller and denser than those of Western women (18). In addition, the incidence of breast cancer onset peaks at 40–50 years in Chinese women, i.e., 5–10 years younger than in their Western counterparts (18). Therefore, developing screening guidelines based on the Chinese population is crucial. Some breast cancer screening programs are being developed in China (35). China is a vast country with a large population, and cancer screening programs were historically implemented in specific areas in the 1980s, mainly Shanghai and Beijing areas, with smaller cities and rural areas implementing their programs later (36). Nevertheless, the idea of breast cancer screening has been public knowledge for a long time despite the fact that access was not incorporated into the basic public health services until 2019 (36).

Since most Chinese women have dense breasts, ultrasound has a better detection value than mammography (37). Beyond basic breast cancer screening, the present study showed that the participants had a good KAP toward breast cancer and screening using ultrasound. Of note, the participants had a relatively high socioeconomic status (i.e., high education, employed, and urban residents), and it is well-known that health literacy is related to socioeconomic status (38). Previous studies performed in the United States of America (27, 39), Saudi Arabia (40), and Macao (41) showed relatively poor enthusiasm toward ultrasound screening. Except for the study by Gan et al. (41), the other studies were performed in populations with generally less dense breasts in which mammography is generally more suitable as the first-line screening modality (5).

Higher knowledge scores were associated with higher education, as supported by the association of a better socioeconomic status with higher health literacy (38). A higher socioeconomic status is also associated with easier access to healthcare information, either because of easier access to healthcare professionals or reliable sources of information or knowing where to search for reliable information (42). Furthermore, socioeconomic status influences health-seeking behaviors, with people with a lower socioeconomic status visiting the emergency departments and primary physicians and people with a higher status visiting more specialists, buying more prescription drugs, and undergoing more imaging examinations (43). The individuals also have the feeling that their socioeconomic status influences their healthcare (44). The same is seen for breast cancer screening, with a higher likelihood of participating in screening in women with a higher socioeconomic status than those with a lower status (45, 46). As shown by the SEM analysis and supported by the multivariable analyses, knowledge was also related to attitude, which influenced practice, as supported by the KAP theory, which states that knowledge is the basis for attitude and knowledge, while attitude is the force driving practice (20, 21). A history of breast ultrasound was also independently associated with better knowledge and attitude scores, probably because the women sought information or received more information regarding breast ultrasound.

In the present study, the knowledge items that should be improved included the women at risk of breast cancer, the no-radiation nature of ultrasound, the best breast cancer screening interval, the impact of a family history of cancer on a woman’s risk, and the warning symptoms of breast cancer. Some of these items are not specific to ultrasound screening, highlighting that general knowledge about breast cancer screening should be improved. Since knowledge was directly correlated to attitudes and practice, improving these relatively crucial points toward breast cancer in general and breast ultrasound should also translate into even better attitudes and practice. Educational material and interventions should be designed on breast cancer screening and breast ultrasound. Several interventions to improve the knowledge on breast cancer screening have been reported [22 interventions reviewed by Noman et al. (47)], but the interventions were highly heterogeneous, probably because of the heterogeneity in the study populations in terms of socioeconomic status, access to healthcare, healthcare systems, and public health education in general. A recent study highlighted the importance of community-based education programs to improve breast cancer screening and decrease anxiety related to screening (48). The bottom line should be that such education programs must be tailored to the specific population being targeted.

This study had limitations. The participants were from a single center, limiting the number of women recruited and limiting the representativeness of the general population. The results represent the KAP of women in Zhongshan, but whether similar results can be observed elsewhere in China remains to be determined. The participants were recruited by convenience sampling, which may have introduced bias into the study. The distribution of questionnaires within breast specialist facilities introduced a potential bias in the sample toward individuals who were already utilizing healthcare services. The potential for non-representativeness of the general population among participants who voluntarily present themselves at a hospital could restrict the external validity of the findings. Indeed, in the present study, most women enjoyed a higher socioeconomic status than the general population, limiting the generalizability of the conclusions. In addition, this study was cross-sectional, and no conclusion on causality could be made. The present study used a SEM analysis to obtain an estimate of causality, but it must be remembered that a SEM analysis statistically infers causality based on prespecified hypotheses, and the results (although useful for future studies) must be taken with caution (28–30). No previous KAP data were available from the same population, limiting the possible comparisons. Still, the present study could be a kind of baseline to evaluate the impact of future interventions. Nevertheless, studies should first identify the critical knowledge deficits or attitudes that would necessitate improvements. Finally, all KAP surveys are at risk of social desirability bias, in which the participants can answer what they should do instead of what they are doing (49, 50).

## 5 Conclusion

In conclusion, the results suggest that women in Zhongshan have good knowledge, favorable attitudes, and active practice toward breast ultrasound, but this study identified specific knowledge and attitude items that might require improvements. Education interventions should be carried out to improve the KAP of women toward ultrasound breast cancer screening.

## Data availability statement

The original contributions presented in the study are included in the article/Supplementary material, further inquiries can be directed to the corresponding author.

## Ethics statement

The studies involving humans were approved by Medical Ethics Committee of Zhongshan People’s Hospital. The studies were conducted in accordance with the local legislation and institutional requirements. The participants provided their written informed consent to participate in this study.

## Author contributions

SL: Conceptualization, Investigation, Writing – original draft. SZ: Conceptualization, Data curation, Formal analysis, Methodology, Software, Writing – review & editing. MQ: Conceptualization, Data curation, Formal analysis, Methodology, Software, Writing – review & editing. YX: Data curation, Formal analysis, Writing – original draft. KY: Data curation, Formal analysis, Writing – original draft. XL: Conceptualization, Funding acquisition, Investigation, Project administration, Resources, Supervision, Validation, Visualization, Writing – original draft, Writing – review & editing.
