# Supplementary material for: Knowledge, attitude, and practice toward ultrasound screening for breast cancer among women
Source: Front Public Health. 2024 May 24;12:1309797. doi: 10.3389/fpubh.2024.1309797 (PMC11160319; doi:10.3389/fpubh.2024.1309797)
Supplement: Supplementary file 1 [file Table_1.DOCX]

| **Knowledge, attitude, and practice toward ultrasound screening for breast cancer among women** | | |  |
| --- | --- | --- | --- |
| **Part I Basic Information** | | |  |
| **1.Your age** | | a. <30  b. 30-39  c. ≥40 |  |
| **2.** **Residence** | | a. Non-urban  b. Urban |  |
| **3.** **Marital status** | | a. Unmarried  b. Married  c. Divorced  d. Widowed |  |
| **4.** **Education** | | a. Junior high school and below  b. Senior high school  c. Bachelor and above |  |
| **5.** **Fertility status** | | a. Childbearing  b. No pregnancy  c. Pregnancy but not given birth |  |
| **6.** **Working status** | | | a. Employed  b. Housewife or unemployed |
| **7.** **Monthly per capita income, yuan** | | a. < 5000  b. 5000-10000  c. >10,000 |  |
| **8.** **Family history of breast cancer** | | a. Yes  b. No |  |
| **9.** **Breast ultrasound** | | a. Yes  b. No |  |

**Part II Knowledge**

| **1. Breast cancer is the most common malignant tumor that seriously threatens women’s health.** | a. Correct | b. Wrong | c. Unclear |
| --- | --- | --- | --- |
| **2. The age at which women are susceptible to breast cancer.** | a. 18-35 years  b. 35-45 years  c. >45 years  d. All of the above | | |
| **3. The optimal interval for regular breast cancer screening in healthy women.** | a. Once every six months  b. Once every 1-2 years  c. Once every 3 years  d. Once every 5 years | | |
| **4. The pre-symptoms of breast cancer.** | a. Breast mass appears  b. Non pregnancy nipple discharge  c. Breast skin shows small indentations  d. Breast lobular hyperplasia | | |
| **5. Breast cancer can be detected early through breast screening.** | a. Correct | b. Wrong | c. Unclear |
| **6. You have heard of a test called breast ultrasound** | a. Yes | b. No |  |
| **7. Breast ultrasound has radiation like an X-ray.** | a. Correct | b. Wrong | c. Unclear |
| **8. Breast ultrasound can screen for breast cancer.** | a. Correct | b. Wrong | c. Unclear |
| **9. Breast ultrasound is only important for women over 50.** |  |  |  |
| **10. Breast ultrasound is only important for women with a family history of breast cancer.** | a. Correct | b. Wrong | c. Unclear |
| **11. Besides the doctor or their palpation examination, women should also have a breast ultrasound.** | a. Correct | b. Wrong | c. Unclear |
| **12. Women aged 41-70 should have a breast ultrasound at least once a year.** | a. Correct | b. Wrong | c. Unclear |

**Part III Attitude**

| **1. I was very afraid that I would get breast cancer.** | a. Strongly agree | b. Agree | c. Neutral | d. Disagree | e. Strongly disagree |
| --- | --- | --- | --- | --- | --- |
| **2. I think early detection, early diagnosis, and early treatment are very important for breast cancer prevention.** | a. Strongly agree | b. Agree | c. Neutral | d. Disagree | e. Strongly disagree |
| **3. I do a breast ultrasound only because my doctor has already booked an appointment.** | a. Strongly agree | b. Agree | c. Neutral | d. Disagree | e. Strongly disagree |
| **4. If the doctor didn’t recommend it, I wouldn’t do a breast ultrasound.** | a. Strongly agree | b. Agree | c. Neutral | d. Disagree | e. Strongly disagree |
| **5. A breast ultrasound changes my chances of finding a lump before I can feel it.** | a. Strongly agree | b. Agree | c. Neutral | d. Disagree | e. Strongly disagree |
| **6. Having a breast ultrasound once a year will make me feel very at ease.** | a. Strongly agree | b. Agree | c. Neutral | d. Disagree | e. Strongly disagree |
| **7. I think breast ultrasound can detect breast cancer that cannot be detected by mammograms alone.** | a. Strongly agree | b. Agree | c. Neutral | d. Disagree | e. Strongly disagree |
| **8. I want to know more about breast cancer and breast screening.** | a. Strongly agree | b. Agree | c. Neutral | d. Disagree | e. Strongly disagree |

**Part IV Practice**

| **1. If there is a seminar related to breast cancer and screening, I would like to attend.** | a. Strongly agree | b. Agree | c. Neutral | d. Disagree | e. Strongly disagree |
| --- | --- | --- | --- | --- | --- |
| **2. I want to know if I’m at high risk for breast cancer.** | a. Strongly agree | b. Agree | c. Neutral | d. Disagree | e. Strongly disagree |
| **3. I plan to have a breast ultrasound at least once a year.** | a. Strongly agree | b. Agree | c. Neutral | d. Disagree | e. Strongly disagree |
| **4. If a friend/relative recommends a breast ultrasound to me, I will do it** | a. Strongly agree | b. Agree | c. Neutral | d. Disagree | e. Strongly disagree |
| **5. If I know someone has been diagnosed with breast cancer, I will make an appointment for a breast ultrasound as soon as possible.** | a. Strongly agree | b. Agree | c. Neutral | d. Disagree | e. Strongly disagree |
| **6. If I am not unwell, I will not go for a breast ultrasound.** | a. Strongly agree | b. Agree | c. Neutral | d. Disagree | e. Strongly disagree |
| **7. If the last breast ultrasound test is negative, I will relax my vigilance for breast diseases.** | a. Strongly agree | b. Agree | c. Neutral | d. Disagree | e. Strongly disagree |
| **8. Between the two screenings, I will always pay attention to the breast glands and seek medical attention in time if abnormalities are found.** | a. Strongly agree | b. Agree | c. Neutral | d. Disagree | e. Strongly disagree |
